# Supplementary material for: Curated and harmonised transcriptomics datasets of interstitial lung diseases
Source: Data Brief. 2025 Oct 14;63:112139. doi: 10.1016/j.dib.2025.112139 (PMC12581653; doi:10.1016/j.dib.2025.112139)
Supplement: Supplementary file 1 [file mmc1.zip › Supplementary_material/RNA-seq/fastQC reports/GSE138283/SRR10212999_fastqc.html]

SRR10212999.fastq.gz FastQC Report 

FastQC Report

Fri 2 Feb 2024  
SRR10212999.fastq.gz

## Summary

- Basic Statistics
- Per base sequence quality
- Per sequence quality scores
- Per base sequence content
- Per sequence GC content
- Per base N content
- Sequence Length Distribution
- Sequence Duplication Levels
- Overrepresented sequences
- Adapter Content

## Basic Statistics

| Measure | Value |
| --- | --- |
| Filename | SRR10212999.fastq.gz |
| File type | Conventional base calls |
| Encoding | Sanger / Illumina 1.9 |
| Total Sequences | 92383985 |
| Sequences flagged as poor quality | 0 |
| Sequence length | 35-76 |
| %GC | 50 |

## Per base sequence quality

## Per sequence quality scores

## Per base sequence content

## Per sequence GC content

## Per base N content

## Sequence Length Distribution

## Sequence Duplication Levels

## Overrepresented sequences

| Sequence | Count | Percentage | Possible Source |
| --- | --- | --- | --- |
| CCTTAGGCAACCTGGTGGTCCCCCGCTCCCGGGAGGTCACCATATTGATG | 309031 | 0.33450711181164133 | No Hit |
| CTCCGTTTCCGACCTGGGCCGGTTCACCCCTCCTTAGGCAACCTGGTGGT | 275337 | 0.2980354224815048 | No Hit |
| CCCCTCCTTAGGCAACCTGGTGGTCCCCCGCTCCCGGGAGGTCACCATAT | 222889 | 0.24126367789828507 | No Hit |
| CCAGGCTGGAGTGCAGTGGCTATTCACAGGCGCGATCCCACTACTGATCA | 216354 | 0.23418994103794072 | No Hit |
| CTTCGATGTCGGCTCTTCCTATCATTGTGAAGCAGAATTCACCAAGCGTT | 196032 | 0.2121926219138523 | No Hit |
| CCCTCCTTAGGCAACCTGGTGGTCCCCCGCTCCCGGGAGGTCACCATATT | 174885 | 0.1893022908678382 | No Hit |
| GTTGGATTGTTCACCCACTAATAGGGAACGTGAGCTGGGTTTAGACCGTC | 164721 | 0.17830038398971423 | No Hit |
| CTCAGGCTGGAGTGCAGTGGCTATTCACAGGCGCGATCCCACTACTGATC | 160385 | 0.1736069298158117 | No Hit |
| GGGAGTTTGACTGGGGCGGTACACCTGTCAAACGGTAACGCAGGTGTCCT | 154403 | 0.16713178155283084 | No Hit |
| CAGGCTGGAGTGCAGTGGCTATTCACAGGCGCGATCCCACTACTGATCAG | 123234 | 0.13339324992313334 | No Hit |
| CTCCTTAGGCAACCTGGTGGTCCCCCGCTCCCGGGAGGTCACCATATTGA | 121451 | 0.1314632617330807 | No Hit |
| CTTAGGCAACCTGGTGGTCCCCCGCTCCCGGGAGGTCACCATATTGATGC | 115728 | 0.12526846509164982 | No Hit |
| CCTCCTTAGGCAACCTGGTGGTCCCCCGCTCCCGGGAGGTCACCATATTGATGCCGAACTTAGTGCGGACACCCG | 113253 | 0.12258942932587288 | No Hit |
| GCTCCGTTTCCGACCTGGGCCGGTTCACCCCTCCTTAGGCAACCTGGTGGTCCCCCGCTCCCGGGAGGTCACCAT | 98507 | 0.10662778835530855 | No Hit |
| GTCGGCTCTTCCTATCATTGTGAAGCAGAATTCACCAAGCGTTGGATTGT | 97370 | 0.10539705556109104 | No Hit |

## Adapter Content

Produced by FastQC (version 0.11.7)
